# Comprehensive Analysis of Pediatric Supracondylar Fractures in the Emergency Department; A Single Center Experience

**DOI:** 10.30476/BEAT.2020.83195

**Published:** 2020-07

**Authors:** José Ramón Ausó-Pérez, Gloria María Rodríguez-Blanes

**Affiliations:** 1 *Orthopedic Surgery and Traumatology Services, Hospital Marina Baixa, Alicante, Spain*; 2 *Occupational Health Unit, Public Health Center of Alcoy, Alicante; Conselleria of Universal Health and Public Health, Generalitat Valenciana, Spain*

**Keywords:** Supracondylar, Elbow, Pediatric, Epidemiology, Seasonal

## Abstract

**Objective:**

To describe the demographic characteristics and to assess possible risk factors related to the moment of presentation at Emergency Department (ED) for pediatric humeral supracondylar fractures.

**Methods:**

This was cross-sectional study being conducted during 5-year period from 2013 to 2017 at ED of a regional hospital in Spain. We have included all the pediatric patients (<14 years) with supracondylar fractures referring to our center during the study period. The demographic, clinical and radiological characteristics of the patients were recorded. The outcome and treatment strategies were also recorded. The risk factors of the pediatric supracondylar fracture was also assessed in our series.

**Results:**

We have included 52 pediatric patients with supracondylar fractures in this series. The mean age was 7.48 ± 2.97 years with a minimum age of 2 years and a maximum of 14 years. Among the patients there were 32 (61.54%) male and 20 (38.46%) female. Age less than 7 years found to be a protective factor against unstable fractures [OR 0.33 (0.10 - 1.02)]. Fractures presented during daytime hours showed a greater instability [OR 3.49 (1.07-11.39)]. However, the risk of presentation at nighttime was higher during the summer months (June to September).

**Conclusion:**

The older is the child, the greater is the risk of suffering an unstable fracture, which increases the need for surgery. This risk is greater during the daytime. Otherwise, during the summer months, there is a higher risk of patient presentation at nighttime.

## Introduction

Supracondylar humeral fracture is the most frequent fracture around the elbow during childhood (55-80%) [1-3]. They are also one of the fractures that require more surgery during the pediatric age [4]. They account for 15% of all fractures in pediatric age and their incidence has been increasing in recent years [3, 5, 6]. The median age at which this injury occurs is reported variably as 3 to 8 years in different studies [5, 7]. Although a greater incidence in males has traditionally been indicated, recent studies do not find differences regarding gender [5, 8-10].

Most of these fractures occur after falls with the elbow in extension [7, 8, 11, 12]. Several studies have shown variations in the time of year in which these fractures occur, indicating a peak during the summer months [7, 9, 11]. However, few studies have studied whether there is a predominant time pattern in this type of fracture [13, 14]. Taking into account the controversy about the opportune moment of the treatment of this type of fractures, this factor could be of great importance and utility to dedicate resources in the Emergencies Department for the initial attention as well as for the need of operating resources [15-17]. Likewise, recreational and sports activities carried out during childhood have varied in recent years [10]. Therefore, updating epidemiological data is something that should be done periodically. This study aims to describe the characteristics of patients diagnosed for supracondylar humerus fractures in the Emergency Department (ED) of a Regional Hospital and to study possible associations between those characteristics with the type of fracture (stable or unstable) or the moment of presentation in the ED (day and hour).

## Materials and Methods

*Study population*

A retrospective, cross-sectional study was carried out including all patients under 14 years of age diagnosed with a humeral supracondylar fracture presenting to the ED of a Regional Hospital in Alicante (Spain) during the years 2013 to 2017. It was not taken into account whether the patient was operated on or not. Patients with incomplete medical records were not included. The study protocol was approved by the institutional review board (IRB) and medical ethics committee of our hospital. As this was retrospective chart review. No informed written consent was required for the study. 

*Study protocol*

We selected all cases diagnosed in the Emergencies Department with codes ICD-9 812 and ICD-10 S42, with all the subcodes. Lately, all cases were reviewed and those that did not correspond with a supracondylar fracture, such as epicondyle or unicondylar fractures, were excluded. Radiographs of all selected cases were reviewed to classify each fracture according to the Gartland classification. For type II, we divided the fractures in those without rotation (type IIA) o rotated (type IIB) [12]. The data of these patients were obtained in cooperation with the Archive and Documentation Service of the Hospital. To guarantee confidentiality and anonymity, the personal identification data were eliminated, and a consecutive reference number to each case was assigned. Throughout the process, the ethics and quality standards established by the Regional Government (Consellería de Sanidad) were respected. Being a descriptive observational study that does not imply any intervention on patients and that did not need to contact any patient to obtain complementary data, it was not necessary to obtain any additional consent. The variables studied were demographic (age of the patients at the time of the fracture and gender of each patient) and characteristics of the fracture (date and hour of admission to the Emergencies Department of the Hospital, laterality of the fracture and type of fracture according to the Gartland classification). After the first coding, variables were grouped into dichotomous groups. The time was grouped into day, as entered between 8:00 a.m. and 8:00 p.m., or night, later 8:00 p.m. The date was recorded according to the month in summer (between June and September) and the rest of the year. The day was classified according to whether it was a working day (Monday to Friday) or holiday/weekend.

*Statistical analysis*

The qualitative variables were described by the absolute and relative frequency in percentages of each of the values of the variables. The only quantitative variable (age) was described by the mean and the standard deviation as dispersion measures. The study of associations between qualitative variables was carried out through a bivariate analysis using the Chi-square test. To quantify the magnitude of the association between qualitative variables, the Odds Ratio (OR) with a 95% confidence interval was calculated. The level of statistical significance used in the contrasts of hypotheses has been 0.05 and a p-value of less than 0.05 was considered statistically significant.

## Results

A total of 52 patients under 14 years of age with a diagnosis of distal humorous supracondylar fracture were obtained (Table 1). Among the patients there were 32 (61.54%) male and 20 (38.46%) female. The most affected side was the left, corresponding to 67.31% (n=35). The mean age was 7.48 (± 2.97) years with a minimum age of 2 years and a maximum of 14 years. Among the patients, 50.0% were older than 7 years. Regarding the moment of the fracture, 57.69% (n=30) occurred between 8:00 A.M. and 8:00 P.M., while 48.08% (n=25) occurred in summer. About, 67.31% (n=35) of the fractures took place on a working day (Table 1). 

According to this, Gartland type I fractures were the most frequent, corresponding to 38.46% (n = 20) of the total (Table 1). After the bivariate analysis, children under 7 years old had a lower number of unstable fractures [OR 0.33 (0.10 -1.02)]. Fractures that occurred during daytime hours showed higher instability [OR 3.49 (1.07-11.39)]. No differences were observed in the rest of the factors studied (Table 2). The multivariate analysis confirms the results obtained about the increased risk of suffering an unstable fracture during daytime hours (from 8:00 am to 8:00 pm) compared to nighttime, showing itself as an independent risk factor (Table 2). There was also a higher risk of a fracture occurring after 8:00 pm during the summer months (June to September) with an OR of 4.29 (1.32-13.88) (Table 3). The multivariate analysis confirms that the summer season behaves as an independent risk factor as itself for presenting a supracondylar fracture (Table 3). 

## Discussion

Supracondylar fractures are the most common elbow fractures during childhood, they accounted for about 18% of all pediatric fractures [2, 11]. The major part of them have been associated with falls with the arm in extension (90-95%) and affected the non-dominant side [2, 6, 11]. According to our results, we observed a greater number of fractures in boys compared to girls, a finding consistent with numerous studies [6, 11, 12]. However, no differences were found regarding the characteristics or timing of presentation in both genders. These results are similar to those obtained in the most recent studies of the demography of this type of fracture, compared to the classic results that indicated a higher frequency in males [6-8, 10]. A possible explanation for this change in the trend could be due to the emergence of a more equality culture in terms of education and games [10]. The average age obtained in our patients (7.49 years) is similar to the range observed in other studies ranging between 4 and 9 years [6, 9, 11]. The left side was the most affected, which agrees with the literature [7, 8].

Although the literature indicates that the non-dominant side suffers this type of fracture more frequently [7,8,11,12], this data was not taken into account in this study due to the lack of this data in most of the revised ER reports. In terms of the type of fracture, two results stand out in the statistical analysis. On the one hand, it is observed that age less than or equal to 7 years is shown as a protective factor against unstable fracture (types IIB and III of Gartland) [OR 0.33 (0.10-1.02)]. However, the multivariate analysis does not confirm as an independent effect (Table 2). Recent literature indicates data in line with the results obtained in our study [18]. On the other hand, the fact that the patient comes to Emergencies Department during the day (before 8:00 p.m.) is a risk factor for the fracture to be unstable both in the bivariate as in the multivariate analysis [OR 7.17 (1.45 – 35.55)]. Several studies indicate that outdoor activities and sports activities increase the risk of more severe fractures [7, 10]. Most of these activities are carried out during daytime hours, as well as in school, which could be a plausible explanation for this point, and even some studies have shown that the modification of playground surfaces could decrease the frequency of fractures and their costs [4, 19].

Regarding the moment of the presentation of the patient, according to our results, and despite not finding differences neither in gender, affected side or age, we do observe differences in the type of fracture, and above all, in the season of the year when it happens. According to our results, the probability of a fracture occurring at night (beyond 8:00 p.m.) is 4.29 times higher in summer months (June to September) than during the rest of the year. This result agrees with numerous studies that indicate that the risk of producing a supracondylar fracture is higher during the summer months [2, 6, 7, 9, 11, 13]. Some studies have studied the relationship between meteorological conditions and the incidence of supracondylar fractures [3, 13, 14]. In them, it was indicated that during the sunny days and with warmer temperatures, the incidence increased [3]. However, very few studies have studied the time of the day when patient care is provided [14]. One study observed a peak of fractures at the end of the day, and that in places with a more stable temperature and longer hours of Sun the peak incidence of fractures was delayed at later hours [14]. The reference area of the Hospital from which the data were obtained corresponds to a touristic region, especially during summertime, with familiar tourism and which presents mild temperatures and more hours of sunshine during summertime. Therefore, the sum of factors such as relatively warm temperatures, the nearby of the sea, staying more time outside the house/hotel and some relaxation in the monitoring of the children could cause the increase in the incidence of fractures, which is similar to the observed in several previous works [3, 11, 13, 14].

Knowing the most frequent time and season of the presentation of cases can also be useful to rationalize the means and the planning of surgery in the cases that it could be necessary [8, 11]. Recent studies have shown that complications are higher in those patients operated outside office hours [1, 17]. However, the American Academy of Orthopaedic Surgeons does not provide specific indications on this point [15]. Our study shows that the majority of unstable fractures, which therefore required surgical treatment, were presented before 8:00 p.m. This can cause the time of surgery to be delayed even up to 12 hours or more. Several studies spoke about the need to reinforce the evening shift to be able to act on these fractures as soon as possible and with the best possible results [14, 16]. Other works, however, recommended carrying out the interventions during daytime hours, except in cases of neurovascular injuries at the time of admission, since they reduced the complications and the final cost of the process [1, 17, 20]. Due to the controversy existing at this point, more studies would be necessary to unify the best criteria for action in each case.

As limitations of this study, we have those of any observational study. As well as the cases are obtained from the records of the Emergencies Department, if there was an error in the coding of the diagnosis, it could have caused the loss of several cases. In the aim to minimize these errors, all the doubtful cases were included in the initial selection and a case-by-case review was carried out to discard and/or rescue all possible coding errors. Also, since it is a tourist zone, not all the cases that have occurred go to the Public Hospital since many patients have private travel insurance, so reliable data for prevalence or incidence study cannot be obtained from our results. This same situation also limits the size of the population studied. Other studies extend both the period of case recruitment and the age of the patients (reaching a period of 10 years and up to 18 years of age) [2].

Another limitation of this study consists of the moment of the presentation of the patients themselves. Probably in the case of patients with minor injuries, parents may think it is just a bruise and present only when the pain persists. So, the moment in which the fracture occurs could be earlier. It is difficult to assess this point, since it depends on a very good anamnesis to the patient or their relatives, for that reason it was decided to use as data only their arrival at the Emergencies Department since this point is the one that most affects the needs for human and material resources.

In conclusion, supracondylar humeral fractures represent one of the fractures that require surgical treatment more frequently during childhood. Older age has an increased risk of needing surgery, as well as those fractures that occur during daylight hours (before 8:00 pm), probably concerning the performance of outdoor and sports activities. During the summer there is a higher risk of fracture in later hours, probably due to more hours of sunshine and milder temperatures. Thus, the hour factor must be considered for the planning of resources, as well as to implement educational strategies for the general population to prevent this type of fracture, especially in younger children.

**Table 1:** Main demographic characteristics of our cases (n=52).

	**%**	**N**
**Total Cases**	100,00%	52
**Gender**		
Male	61,54%	32
Female	38,46%	20
**Side**		
Right	32,69%	17
Left	67,31%	35
**Time Zone**		
Daytime	57,69%	30
Nighttime	42,31%	22
**Station**		
Summertime	48,08%	25
Rest of the year	51,92%	27
**Type of Day**		
Holiday	32,69%	17
Working Day	67,31%	35
**Classification**		
I	38,46%	20
IIA	17,31%	9
IIB	11,54%	6
III	32,69%	17
**Age**		
≤7 years old	50.0%	26
>7 years old	50.0%	26

**Table 2:** Analysis of the association between demographic variables and type of fracture

	**Unstable (n=23)**	**Stable (n=29)**	**OR (CI 95%)**	**p-Value**	**aOR (IC 95%)**	**p-Value**
**Gender**						
Female	43.5% (10)	34.5%(10)	1.46 (0.47 – 4.50)	NS^a^	1.40 (0.35 – 5.66)	NS^a^
Male	56.5% (13)	65.5% (19)	1		1	
**Side**						
Right	30.4% (7)	34.5% (10)	0.83 (0.26 – 2.69)	NS^a^	1.04 (0.25 – 4.37)	NS^ a^
Left	69.6% (16)	65.5% (19)	1			
**Age**						
≤7 years old	34.8 (8)	62.1% (18)	0.33 (0.10 – 1.02)	0.051	0.32 (0.08 – 1.20)	0.090
>7 years old	65.2% (15)	37.9% (11)	1			
**Time Zone**						
Daytime	73.9% (17)	44.8% (13)	3.49 (1.07 – 11.39)	0.035	7.17 (1.45 – 35.55)	0.016
Nighttime	26.1% (6)	55.2% (16)	1			
**Station**						
Summertime	56,5% (13)	41,4% (12)	1.84 (0.61 – 5.57)	NS^a^	3.61 (0.78 – 16.68)	0.100
Rest of the year	43,5% (10)	58,6% (17)	1			
**Type of Day**						
Holiday	21.7% (5)	41.4% (12)	0.39 (0.11 – 1.35)	0.134	0.38 (0.09 – 1.57)	0.180
Working Day	78.3% (18)	58.6% (17)	1			

^a^ **NS:** P> 0.200. Adjusted for: Gender, Side, Age, Time Zone, Season of the year, Type of day.

**Table 3:** Analysis of the association between demographic variables and the moment of the presentation of the patient at Emergencies Department

	**Nighttime (n=23)**	**Daytime (n=29)**	**OR (IC 95%)**	**p-Value**	**aOR (IC 95%)**	**p-Value**
**Gender**						
Male	31.82% (7)	43.33% (13)	0.61 (0.19 – 1.93)	NS^a^	0.59 (0.15 – 2.28)	NS^a^
Female	68.18% (15)	56.67% (17)				
**Side**						
Right	31.82% (7)	33.33% (10)	0.93 (0.29 – 3.02)	NS^a^	1.04 (0.25 – 4.39)	NS^a^
Left	68.18% (15)	66.67% (20)				
**Age**						
≤7 years old	50.00% (11)	50.00% (15)	1.00 (0.33 – 3.01)	NS^a^	0.97 (0.25 – 3.79)	NS^a^
>7 years old	50.00% (11)	50.00% (15)				
**Type of fracture**						
Unstable	27.27% (6)	56.67% (17)	0.29 (0.09 – 0.94)	0.035	0.15 (0.03 – 0.73)	0.019
Stable	72.73% (16)	43.33% (13)				
**Station**						
Summertime	68.18% (15)	33.33% (10)	4.29 (1.32 – 13.88)	0.013	7.96 (1.80 – 35.11)	0.006
Rest of the year	31.82% (7)	66.67% (20)				
**Type Of Day**						
Holiday	36.36% (8)	30.00% (9)	1.33 (0.41 – 4.29)	NS^a^	0.94 (0.23 – 3.93)	NS^a^
Working Day	63.64% (14)	70.00% (21)				

^a^ **NS:** P >0.200; Adjusted for: Gender, Side, Age, Time Zone, Season of the year, Type of day.

## Conflict of Interest:

The authors declare that they have no conflict of interest.

## Sources of funding:

This research did not receive any specific grant from funding agencies in the public, commercial, or not-for-profit sectors.
